# Supplementary material for: A Three-Protein Panel to Support the Diagnosis of Sepsis in Children
Source: J Clin Med. 2022 Mar 12;11(6):1563. doi: 10.3390/jcm11061563 (PMC8955185; doi:10.3390/jcm11061563)
Supplement: Supplementary file 1 [file jcm-11-01563-s001.zip › jcm-1612869-supplementary.pdf]

Supplementary material

Table S1.Proteins identified by proteomics

ACCESSION	ANOVA (P)	MAX FOLD CHANGE	HIGHEST MEAN CONDITION	DESCRIPTION
**LBP_HUMAN**	2,60E-09	29,31263	Sepsis	Lipopolysaccharide-binding protein OS=Homo sapiens GN=LBP PE=1 SV=3
**A2GL_HUMAN**	3,20E-08	4,426432	Sepsis	Leucine-rich alpha-2-glycoprotein OS=Homo sapiens GN=LRG1 PE=1 SV=2
**CRP_HUMAN**	8,14E-08	30,41798	Sepsis	C-reactive protein OS=Homo sapiens GN=CRP PE=1 SV=1
**ZBT10_HUMAN**	2,53E-07	56,29807	Sepsis	Zinc finger and BTB domain-containing protein 10 OS=Homo sapiens GN=ZBTB10 PE=1 SV=2
**SAA2_HUMAN**	7,47E-07	84,27766	Sepsis	Serum amyloid A-2 protein OS=Homo sapiens GN=SAA2 PE=1 SV=1
**IPSP_HUMAN**	1,21E-06	5,010882	Control	Plasma serine protease inhibitor OS=Homo sapiens GN=SERPINA5 PE=1 SV=3
**SAM9L_HUMAN**	1,56E-06	4,739466	Sepsis	Sterile alpha motif domain-containing protein 9-like OS=Homo sapiens GN=SAMD9L PE=1 SV=2
**FINC_HUMAN**	9,13E-06	8,758802	Control	Fibronectin OS=Homo sapiens GN=FN1 PE=1 SV=4
**HBB_HUMAN**	1,11E-05	25,27837	Sepsis	Hemoglobin subunit beta OS=Homo sapiens GN=HBB PE=1 SV=2
**AACT_HUMAN**	1,88E-05	5,283651	Sepsis	Alpha-1-antichymotrypsin OS=Homo sapiens GN=SERPINA3 PE=1 SV=2
**VASN_HUMAN**	2,50E-05	2,193836	Sepsis	Vasorin OS=Homo sapiens GN=VASN PE=1 SV=1
**SAA1_HUMAN**	2,97E-05	76,09709	Sepsis	Serum amyloid A-1 protein OS=Homo sapiens GN=SAA1 PE=1 SV=1
**HBA_HUMAN**	3,78E-05	16,39969	Sepsis	Hemoglobin subunit alpha OS=Homo sapiens GN=HBA1 PE=1 SV=2
**CATA_HUMAN**	5,92E-05	7,286451	Sepsis	Catalase OS=Homo sapiens GN=CAT PE=1 SV=3
**HABP2_HUMAN**	0,000159	2,179075	Control	Hyaluronan-binding protein 2 OS=Homo sapiens GN=HABP2 PE=1 SV=1
**TTHY_HUMAN**	0,000215	2,365905	Control	Transthyretin OS=Homo sapiens GN=TTR PE=1 SV=1
**CAH1_HUMAN**	0,000234	8,950114	Sepsis	Carbonic anhydrase 1 OS=Homo sapiens GN=CA1 PE=1 SV=2
**EZRI_HUMAN**	0,000299	13,5159	Sepsis	Ezrin OS=Homo sapiens GN=EZR PE=1 SV=4
**CFAB_HUMAN**	0,000336	2,048895	Sepsis	Complement factor B OS=Homo sapiens GN=CFB PE=1 SV=2
**HBG1_HUMAN**	0,00038	10,34474	Sepsis	Hemoglobin subunit gamma-1 OS=Homo sapiens GN=HBG1 PE=1 SV=2
**LYAM1_HUMAN**	0,0004	2,675523	Sepsis	L-selectin OS=Homo sapiens GN=SELL PE=1 SV=2
**NGAL_HUMAN**	0,000557	7,169257	Sepsis	Neutrophil gelatinase-associated lipocalin OS=Homo sapiens GN=LCN2 PE=1 SV=2
**BGH3_HUMAN**	0,000634	3,273124	Sepsis	Transforming growth factor-beta-induced protein ig-h3 OS=Homo sapiens GN=TGFBI PE=1 SV=1
**B2MG_HUMAN**	0,000753	3,486171	Sepsis	Beta-2-microglobulin OS=Homo sapiens GN=B2M PE=1 SV=1
**KAIN_HUMAN**	0,000763	1,80505	Control	Kallistatin OS=Homo sapiens GN=SERPINA4 PE=1 SV=3
**FA12_HUMAN**	0,00083	3,237085	Control	Coagulation factor XII OS=Homo sapiens GN=F12 PE=1 SV=3
**CD14_HUMAN**	0,000871	2,784851	Sepsis	Monocyte differentiation antigen CD14 OS=Homo sapiens GN=CD14 PE=1 SV=2
**FIBG_HUMAN**	0,000995	5,317049	Sepsis	Fibrinogen gamma chain OS=Homo sapiens GN=FGG PE=1 SV=3
**TRFL_HUMAN**	0,001181	9,346009	Sepsis	Lactotransferrin OS=Homo sapiens GN=LTF PE=1 SV=6
**ITIH3_HUMAN**	0,001239	2,192084	Sepsis	Inter-alpha-trypsin inhibitor heavy chain H3 OS=Homo sapiens GN=ITIH3 PE=1 SV=2
**SAP_HUMAN**	0,001275	15,28159	Sepsis	Prosaposin OS=Homo sapiens GN=PSAP PE=1 SV=2
**GPX3_HUMAN**	0,001346	1,841343	Sepsis	Glutathione peroxidase 3 OS=Homo sapiens GN=GPX3 PE=1 SV=2
**DEF1_HUMAN**	0,001388	3,067017	Sepsis	Neutrophil defensin 1 OS=Homo sapiens GN=DEFA1 PE=1 SV=1
**PRDX2_HUMAN**	0,00188	7,267809	Sepsis	Peroxiredoxin-2 OS=Homo sapiens GN=PRDX2 PE=1 SV=5
**LIRA1_HUMAN**	0,001982	19,79422	Sepsis	Leukocyte immunoglobulin-like receptor subfamily A member 1 OS=Homo sapiens GN=LILRA1 PE=2 SV=1
**A1AG1_HUMAN**	0,002063	2,94118	Sepsis	Alpha-1-acid glycoprotein 1 OS=Homo sapiens GN=ORM1 PE=1 SV=1
**FIBB_HUMAN**	0,002142	7,926361	Sepsis	Fibrinogen beta chain OS=Homo sapiens GN=FGB PE=1 SV=2

**APOA4_HUMAN**	0,002251	3,062538	Control	Apolipoprotein A-IV OS=Homo sapiens GN=APOA4 PE=1 SV=3
**RET4_HUMAN**	0,002343	2,804231	Control	Retinol-binding protein 4 OS=Homo sapiens GN=RBP4 PE=1 SV=3
**CSF1R_HUMAN**	0,002473	2,273012	Sepsis	Macrophage colony-stimulating factor 1 receptor OS=Homo sapiens GN=CSF1R PE=1 SV=2
**PLMN_HUMAN**	0,002495	2,124858	Control	Plasminogen OS=Homo sapiens GN=PLG PE=1 SV=2
**ITIH2_HUMAN**	0,003087	1,890549	Control	Inter-alpha-trypsin inhibitor heavy chain H2 OS=Homo sapiens GN=ITIH2 PE=1 SV=2
**MA1A1_HUMAN**	0,003474	17,1192	Sepsis	Mannosyl-oligosaccharide 1,2-alpha-mannosidase IA OS=Homo sapiens GN=MAN1A1 PE=1 SV=3
**G3P_HUMAN**	0,004421	3,421498	Sepsis	Glyceraldehyde-3-phosphate dehydrogenase OS=Homo sapiens GN=GAPDH PE=1 SV=3
**SHBG_HUMAN**	0,004819	2,27438	Sepsis	Sex hormone-binding globulin OS=Homo sapiens GN=SHBG PE=1 SV=2
**SPRC_HUMAN**	0,005756	2,146585	Control	SPARC OS=Homo sapiens GN=SPARC PE=1 SV=1
**HGFL_HUMAN**	0,006618	1,904603	Sepsis	Hepatocyte growth factor-like protein OS=Homo sapiens GN=MST1 PE=1 SV=2
**APOH_HUMAN**	0,006849	1,769107	Control	Beta-2-glycoprotein 1 OS=Homo sapiens GN=APOH PE=1 SV=3
**REG1A_HUMAN**	0,007276	6,428111	Sepsis	Lithostathine-1-alpha OS=Homo sapiens GN=REG1A PE=1 SV=3
**FETUB_HUMAN**	0,007872	2,061183	Control	Fetuin-B OS=Homo sapiens GN=FETUB PE=1 SV=2
**HPT_HUMAN**	0,008058	9,390239	Sepsis	Haptoglobin OS=Homo sapiens GN=HP PE=1 SV=1
**PTX3_HUMAN**	0,008478	8,831504	Sepsis	Pentraxin-related protein PTX3 OS=Homo sapiens GN=PTX3 PE=1 SV=3
**6PGD_HUMAN**	0,008556	3,129853	Sepsis	6-phosphogluconate dehydrogenase, decarboxylating OS=Homo sapiens GN=PGD PE=1 SV=3
**CLUS_HUMAN**	0,008586	1,578308	Control	Clusterin OS=Homo sapiens GN=CLU PE=1 SV=1
**AMBP_HUMAN**	0,010741	1,541423	Control	Protein AMBP OS=Homo sapiens GN=AMBP PE=1 SV=1
**ZPI_HUMAN**	0,011272	2,79504	Sepsis	Protein Z-dependent protease inhibitor OS=Homo sapiens GN=SERPINA10 PE=1 SV=1
**TRFE_HUMAN**	0,011976	1,689484	Control	Serotransferrin OS=Homo sapiens GN=TF PE=1 SV=3
**KLKB1_HUMAN**	0,011997	1,515679	Control	Plasma kallikrein OS=Homo sapiens GN=KLKB1 PE=1 SV=1
**RIMS1_HUMAN**	0,013036	1,840342	Control	Regulating synaptic membrane exocytosis protein 1 OS=Homo sapiens GN=RIMS1 PE=1 SV=1
**CO9_HUMAN**	0,015303	1,736343	Sepsis	Complement component C9 OS=Homo sapiens GN=C9 PE=1 SV=2
**APOA1_HUMAN**	0,016232	1,736786	Control	Apolipoprotein A-I OS=Homo sapiens GN=APOA1 PE=1 SV=1
**COR1A_HUMAN**	0,016806	4,218491	Sepsis	Coronin-1A OS=Homo sapiens GN=CORO1A PE=1 SV=4
**PLSL_HUMAN**	0,016815	2,812893	Sepsis	Plastin-2 OS=Homo sapiens GN=LCP1 PE=1 SV=6
**VTNC_HUMAN**	0,017155	1,495985	Sepsis	Vitronectin OS=Homo sapiens GN=VTN PE=1 SV=1
**MA2A1_HUMAN**	0,017246	2,095398	Sepsis	Alpha-mannosidase 2 OS=Homo sapiens GN=MAN2A1 PE=1 SV=2
**1433Z_HUMAN**	0,017304	9,387205	Sepsis	14-3-3 protein zeta/delta OS=Homo sapiens GN=YWHAZ PE=1 SV=1
**THRB_HUMAN**	0,018071	1,567337	Control	Prothrombin OS=Homo sapiens GN=F2 PE=1 SV=2
**APOE_HUMAN**	0,018251	1,630123	Sepsis	Apolipoprotein E OS=Homo sapiens GN=APOE PE=1 SV=1
**APOC3_HUMAN**	0,018703	1,966803	Control	Apolipoprotein C-III OS=Homo sapiens GN=APOC3 PE=1 SV=1
**ACTB_HUMAN**	0,019985	3,024146	Sepsis	Actin, cytoplasmic 1 OS=Homo sapiens GN=ACTB PE=1 SV=1
**CERU_HUMAN**	0,020195	1,556162	Sepsis	Ceruloplasmin OS=Homo sapiens GN=CP PE=1 SV=1
**PZP_HUMAN**	0,020742	1,718804	Control	Pregnancy zone protein OS=Homo sapiens GN=PZP PE=1 SV=4
**CO8A_HUMAN**	0,022202	1,594109	Sepsis	Complement component C8 alpha chain OS=Homo sapiens GN=C8A PE=1 SV=2
**IGHG1_HUMAN**	0,022503	1,817149	Control	Ig gamma-1 chain C region OS=Homo sapiens GN=IGHG1 PE=1 SV=1
**POSTN_HUMAN**	0,023622	2,072017	Sepsis	Periostin OS=Homo sapiens GN=POSTN PE=1 SV=2
**CBPN_HUMAN**	0,02621	2,402578	Sepsis	Carboxypeptidase N catalytic chain OS=Homo sapiens GN=CPN1 PE=1 SV=1
**APOC1_HUMAN**	0,026859	2,093199	Control	Apolipoprotein C-I OS=Homo sapiens GN=APOC1 PE=1 SV=1
**1433E_HUMAN**	0,027517	8,148971	Sepsis	14-3-3 protein epsilon OS=Homo sapiens GN=YWHAE PE=1 SV=1

**LDHA_HUMAN**	0,029135	2,579157	Sepsis	L-lactate dehydrogenase A chain OS=Homo sapiens GN=LDHA PE=1 SV=2
**CD5L_HUMAN**	0,033286	6,760591	Control	CD5 antigen-like OS=Homo sapiens GN=CD5L PE=1 SV=1
**CNDP1_HUMAN**	0,035048	1,931564	Control	Beta-Ala-His dipeptidase OS=Homo sapiens GN=CNDP1 PE=1 SV=4
**ANT3_HUMAN**	0,035467	1,398621	Sepsis	Antithrombin-III OS=Homo sapiens GN=SERPINC1 PE=1 SV=1
**ALDOB_HUMAN**	0,037227	5,740726	Sepsis	Fructose-bisphosphate aldolase B OS=Homo sapiens GN=ALDOB PE=1 SV=2
**CO8B_HUMAN**	0,037467	1,744566	Sepsis	Complement component C8 beta chain OS=Homo sapiens GN=C8B PE=1 SV=3
**K2C1_HUMAN**	0,042086	1,636885	Sepsis	Keratin, type II cytoskeletal 1 OS=Homo sapiens GN=KRT1 PE=1 SV=6
**ALS_HUMAN**	0,044847	1,52681	Control	Insulin-like growth factor-binding protein complex acid labile subunit OS=Homo sapiens GN=IGFALS PE=1 SV=1
**HBD_HUMAN**	0,044995	2,312628	Sepsis	Hemoglobin subunit delta OS=Homo sapiens GN=HBD PE=1 SV=2
**THBG_HUMAN**	0,046199	1,28132	Sepsis	Thyroxine-binding globulin OS=Homo sapiens GN=SERPINA7 PE=1 SV=2
**FA10_HUMAN**	0,047034	1,488008	Sepsis	Coagulation factor X OS=Homo sapiens GN=F10 PE=1 SV=2
**DTNB_HUMAN**	0,047476	4,614486	Sepsis	Dystrobrevin beta OS=Homo sapiens GN=DTNB PE=1 SV=1
**FGL2_HUMAN**	0,049873	8,351645	Sepsis	Fibroleukin OS=Homo sapiens GN=FGL2 PE=1 SV=1
**AFAM_HUMAN**	0,050126	1,560145	Control	Afamin OS=Homo sapiens GN=AFM PE=1 SV=1
**A1AG2_HUMAN**	0,05026	2,118009	Sepsis	Alpha-1-acid glycoprotein 2 OS=Homo sapiens GN=ORM2 PE=1 SV=2
**F16P1_HUMAN**	0,05665	6,770231	Sepsis	Fructose-1,6-bisphosphatase 1 OS=Homo sapiens GN=FBP1 PE=1 SV=5
**FSTL1_HUMAN**	0,057937	6,669486	Sepsis	Follistatin-related protein 1 OS=Homo sapiens GN=FSTL1 PE=1 SV=1
**CCD96_HUMAN**	0,065813	2,655555	Control	Coiled-coil domain-containing protein 96 OS=Homo sapiens GN=CCDC96 PE=2 SV=2
**GP1BA_HUMAN**	0,067618	2,365948	Sepsis	Platelet glycoprotein Ib alpha chain OS=Homo sapiens GN=GP1BA PE=1 SV=2
**PON1_HUMAN**	0,068853	1,502476	Control	Serum paraoxonase/arylesterase 1 OS=Homo sapiens GN=PON1 PE=1 SV=3
**PRG4_HUMAN**	0,070107	1,44814	Sepsis	Proteoglycan 4 OS=Homo sapiens GN=PRG4 PE=1 SV=2
**VCAM1_HUMAN**	0,073323	2,159888	Sepsis	Vascular cell adhesion protein 1 OS=Homo sapiens GN=VCAM1 PE=1 SV=1
**ACTA_HUMAN**	0,075046	2,700992	Sepsis	Actin, aortic smooth muscle OS=Homo sapiens GN=ACTA2 PE=1 SV=1
**CFAI_HUMAN**	0,077141	1,294569	Sepsis	Complement factor I OS=Homo sapiens GN=CFI PE=1 SV=2
**H4_HUMAN**	0,077784	2,759513	Sepsis	Histone H4 OS=Homo sapiens GN=HIST1H4A PE=1 SV=2
**C1R_HUMAN**	0,082565	1,23538	Sepsis	Complement C1r subcomponent OS=Homo sapiens GN=C1R PE=1 SV=2
**VWF_HUMAN**	0,083475	2,0697	Sepsis	von Willebrand factor OS=Homo sapiens GN=VWF PE=1 SV=4
**ITIH1_HUMAN**	0,08811	1,390222	Control	Inter-alpha-trypsin inhibitor heavy chain H1 OS=Homo sapiens GN=ITIH1 PE=1 SV=3
**TKT_HUMAN**	0,089545	11,64935	Sepsis	Transketolase OS=Homo sapiens GN=TKT PE=1 SV=3
**A1BG_HUMAN**	0,09008	1,277178	Sepsis	Alpha-1B-glycoprotein OS=Homo sapiens GN=A1BG PE=1 SV=4
**PGRP2_HUMAN**	0,093453	1,388755	Control	N-acetylmuramoyl-L-alanine amidase OS=Homo sapiens GN=PGLYRP2 PE=1 SV=1
**ALBU_HUMAN**	0,096322	1,450043	Control	Serum albumin OS=Homo sapiens GN=ALB PE=1 SV=2
**FIBA_HUMAN**	0,096837	3,957184	Sepsis	Fibrinogen alpha chain OS=Homo sapiens GN=FGA PE=1 SV=2
**IGHM_HUMAN**	0,097637	2,408566	Control	Ig mu chain C region OS=Homo sapiens GN=IGHM PE=1 SV=3
**SEPP1_HUMAN**	0,098397	1,605897	Control	Selenoprotein P OS=Homo sapiens GN=SEPP1 PE=1 SV=3
**CO7_HUMAN**	0,102856	1,343926	Sepsis	Complement component C7 OS=Homo sapiens GN=C7 PE=1 SV=2
**IGHG3_HUMAN**	0,105844	1,461314	Control	Ig gamma-3 chain C region OS=Homo sapiens GN=IGHG3 PE=1 SV=2
**CFAH_HUMAN**	0,117323	1,17083	Control	Complement factor H OS=Homo sapiens GN=CFH PE=1 SV=4
**KNG1_HUMAN**	0,118254	1,182542	Control	Kininogen-1 OS=Homo sapiens GN=KNG1 PE=1 SV=2
**FCN2_HUMAN**	0,12061	1,325857	Sepsis	Ficolin-2 OS=Homo sapiens GN=FCN2 PE=1 SV=2
**PHLD_HUMAN**	0,126622	1,552526	Control	Phosphatidylinositol-glycan-specific phospholipase D OS=Homo sapiens GN=GPLD1 PE=1 SV=3

**CO4A_HUMAN**	0,126627	1,61536	Sepsis	Complement C4-A OS=Homo sapiens GN=C4A PE=1 SV=2
**A2MG_HUMAN**	0,129819	1,505018	Control	Alpha-2-macroglobulin OS=Homo sapiens GN=A2M PE=1 SV=3
**IGKC_HUMAN**	0,13049	1,604277	Control	Ig kappa chain C region OS=Homo sapiens GN=IGKC PE=1 SV=1
**HEP2_HUMAN**	0,136914	1,286135	Control	Heparin cofactor 2 OS=Homo sapiens GN=SERPIND1 PE=1 SV=3
**IGHG4_HUMAN**	0,139428	1,697464	Control	Ig gamma-4 chain C region OS=Homo sapiens GN=IGHG4 PE=1 SV=1
**HRG_HUMAN**	0,1406	1,327752	Control	Histidine-rich glycoprotein OS=Homo sapiens GN=HRG PE=1 SV=1
**MASP1_HUMAN**	0,141551	1,319306	Control	Mannan-binding lectin serine protease 1 OS=Homo sapiens GN=MASP1 PE=1 SV=3
**TETN_HUMAN**	0,142498	1,311765	Control	Tetranectin OS=Homo sapiens GN=CLEC3B PE=1 SV=3
**K22E_HUMAN**	0,148819	1,160422	Sepsis	Keratin, type II cytoskeletal 2 epidermal OS=Homo sapiens GN=KRT2 PE=1 SV=2
**K1C10_HUMAN**	0,159637	4,744406	Control	Keratin, type I cytoskeletal 10 OS=Homo sapiens GN=KRT10 PE=1 SV=6
**PAR14_HUMAN**	0,181348	1,780101	Control	Poly [ADP-ribose] polymerase 14 OS=Homo sapiens GN=PARP14 PE=1 SV=3
**PI16_HUMAN**	0,182408	1,439091	Control	Peptidase inhibitor 16 OS=Homo sapiens GN=PI16 PE=1 SV=1
**LAC1_HUMAN**	0,184382	2,438967	Control	Ig lambda-1 chain C regions OS=Homo sapiens GN=IGLC1 PE=1 SV=1
**APOM_HUMAN**	0,185041	1,413483	Control	Apolipoprotein M OS=Homo sapiens GN=APOM PE=1 SV=2
**APOC2_HUMAN**	0,187983	1,749555	Control	Apolipoprotein C-II OS=Homo sapiens GN=APOC2 PE=1 SV=1
**CRIS3_HUMAN**	0,189544	2,332398	Sepsis	Cysteine-rich secretory protein 3 OS=Homo sapiens GN=CRISP3 PE=1 SV=1
**CO4B_HUMAN**	0,202706	1,352425	Sepsis	Complement C4-B OS=Homo sapiens GN=C4B PE=1 SV=2
**APOB_HUMAN**	0,205647	1,237763	Control	Apolipoprotein B-100 OS=Homo sapiens GN=APOB PE=1 SV=2
**TSP4_HUMAN**	0,205719	1,565845	Sepsis	Thrombospondin-4 OS=Homo sapiens GN=THBS4 PE=1 SV=2
**CO8G_HUMAN**	0,210404	1,380296	Sepsis	Complement component C8 gamma chain OS=Homo sapiens GN=C8G PE=1 SV=3
**ITIH4_HUMAN**	0,21382	1,270855	Sepsis	Inter-alpha-trypsin inhibitor heavy chain H4 OS=Homo sapiens GN=ITIH4 PE=1 SV=4
**CO3_HUMAN**	0,214364	1,186368	Sepsis	Complement C3 OS=Homo sapiens GN=C3 PE=1 SV=2
**PROS_HUMAN**	0,223306	1,114071	Control	Vitamin K-dependent protein S OS=Homo sapiens GN=PROS1 PE=1 SV=1
**TITIN_HUMAN**	0,231922	1,375777	Control	Titin OS=Homo sapiens GN=TTN PE=1 SV=4
**APOC4_HUMAN**	0,246321	1,536393	Control	Apolipoprotein C-IV OS=Homo sapiens GN=APOC4 PE=1 SV=1
**COMP_HUMAN**	0,259414	2,060502	Sepsis	Cartilage oligomeric matrix protein OS=Homo sapiens GN=COMP PE=1 SV=2
**IGHA1_HUMAN**	0,260265	1,543799	Control	Ig alpha-1 chain C region OS=Homo sapiens GN=IGHA1 PE=1 SV=2
**CETP_HUMAN**	0,26268	1,243085	Sepsis	Cholesteryl ester transfer protein OS=Homo sapiens GN=CETP PE=1 SV=2
**PROC_HUMAN**	0,266888	1,492003	Control	Vitamin K-dependent protein C OS=Homo sapiens GN=PROC PE=1 SV=1
**C1QA_HUMAN**	0,267516	1,217621	Control	Complement C1q subcomponent subunit A OS=Homo sapiens GN=C1QA PE=1 SV=2
**BTD_HUMAN**	0,268922	1,229848	Control	Biotinidase OS=Homo sapiens GN=BTD PE=1 SV=2
**A1AT_HUMAN**	0,27802	2,002863	Sepsis	Alpha-1-antitrypsin OS=Homo sapiens GN=SERPINA1 PE=1 SV=3
**NCAM1_HUMAN**	0,288012	1,349632	Sepsis	Neural cell adhesion molecule 1 OS=Homo sapiens GN=NCAM1 PE=1 SV=3
**ECM1_HUMAN**	0,293942	1,153504	Control	Extracellular matrix protein 1 OS=Homo sapiens GN=ECM1 PE=1 SV=2
**NEO1_HUMAN**	0,29624	2,596158	Sepsis	Neogenin OS=Homo sapiens GN=NEO1 PE=1 SV=2
**HEMO_HUMAN**	0,305632	1,400067	Sepsis	Hemopexin OS=Homo sapiens GN=HPX PE=1 SV=2
**C4BPA_HUMAN**	0,306308	1,172551	Control	C4b-binding protein alpha chain OS=Homo sapiens GN=C4BPA PE=1 SV=2
**LV302_HUMAN**	0,307849	1,453624	Sepsis	Ig lambda chain V-III region LOI OS=Homo sapiens PE=1 SV=1
**LG3BP_HUMAN**	0,32362	1,70481	Sepsis	Galectin-3-binding protein OS=Homo sapiens GN=LGALS3BP PE=1 SV=1
**CO6_HUMAN**	0,325838	1,21449	Sepsis	Complement component C6 OS=Homo sapiens GN=C6 PE=1 SV=3
**ROR1_HUMAN**	0,325897	1,270479	Sepsis	Inactive tyrosine-protein kinase transmembrane receptor ROR1 OS=Homo sapiens GN=ROR1 PE=1 SV=2

**LCAT_HUMAN**	0,328385	1,218859	Control	Phosphatidylcholine-sterol acyltransferase OS=Homo sapiens GN=LCAT PE=1 SV=1
**FA11_HUMAN**	0,33067	1,157884	Control	Coagulation factor XI OS=Homo sapiens GN=F11 PE=1 SV=1
**A2AP_HUMAN**	0,332274	1,187337	Control	Alpha-2-antiplasmin OS=Homo sapiens GN=SERPINF2 PE=1 SV=3
**CYTC_HUMAN**	0,340047	1,114002	Control	Cystatin-C OS=Homo sapiens GN=CST3 PE=1 SV=1
**FBLN1_HUMAN**	0,352373	1,124723	Sepsis	Fibulin-1 OS=Homo sapiens GN=FBLN1 PE=1 SV=4
**PEDF_HUMAN**	0,353736	1,40658	Control	Pigment epithelium-derived factor OS=Homo sapiens GN=SERPINF1 PE=1 SV=4
**UGPA_HUMAN**	0,361997	3,390436	Sepsis	UTP--glucose-1-phosphate uridylyltransferase OS=Homo sapiens GN=UGP2 PE=1 SV=5
**HS90A_HUMAN**	0,366054	4,040204	Sepsis	Heat shock protein HSP 90-alpha OS=Homo sapiens GN=HSP90AA1 PE=1 SV=5
**CPSM_HUMAN**	0,376846	1,405912	Sepsis	Carbamoyl-phosphate synthase [ammonia], mitochondrial OS=Homo sapiens GN=CPS1 PE=1 SV=2
**APOL1_HUMAN**	0,386805	1,322553	Sepsis	Apolipoprotein L1 OS=Homo sapiens GN=APOL1 PE=1 SV=5
**C1S_HUMAN**	0,404196	1,131571	Sepsis	Complement C1s subcomponent OS=Homo sapiens GN=C1S PE=1 SV=1
**K1C9_HUMAN**	0,407161	1,526673	Control	Keratin, type I cytoskeletal 9 OS=Homo sapiens GN=KRT9 PE=1 SV=3
**APOD_HUMAN**	0,410439	1,173274	Control	Apolipoprotein D OS=Homo sapiens GN=APOD PE=1 SV=1
**AL1A1_HUMAN**	0,415011	1,562186	Control	Retinal dehydrogenase 1 OS=Homo sapiens GN=ALDH1A1 PE=1 SV=2
**CAMP_HUMAN**	0,421037	3,167403	Sepsis	Cathelicidin antimicrobial peptide OS=Homo sapiens GN=CAMP PE=1 SV=1
**SAA4_HUMAN**	0,429631	1,201277	Sepsis	Serum amyloid A-4 protein OS=Homo sapiens GN=SAA4 PE=1 SV=2
**FCN3_HUMAN**	0,430777	1,085357	Control	Ficolin-3 OS=Homo sapiens GN=FCN3 PE=1 SV=2
**ANGT_HUMAN**	0,43257	1,490602	Sepsis	Angiotensinogen OS=Homo sapiens GN=AGT PE=1 SV=1
**CPN2_HUMAN**	0,437329	1,352367	Sepsis	Carboxypeptidase N subunit 2 OS=Homo sapiens GN=CPN2 PE=1 SV=3
**GPV_HUMAN**	0,438723	1,081818	Control	Platelet glycoprotein V OS=Homo sapiens GN=GP5 PE=1 SV=1
**C1QB_HUMAN**	0,444358	1,113417	Control	Complement C1q subcomponent subunit B OS=Homo sapiens GN=C1QB PE=1 SV=3
**CBG_HUMAN**	0,446433	1,194661	Sepsis	Corticosteroid-binding globulin OS=Homo sapiens GN=SERPINA6 PE=1 SV=1
**HGFA_HUMAN**	0,487204	1,125065	Control	Hepatocyte growth factor activator OS=Homo sapiens GN=HGFAC PE=1 SV=1
**SCO1_HUMAN**	0,491286	1,075056	Control	Protein SCO1 homolog, mitochondrial OS=Homo sapiens GN=SCO1 PE=1 SV=1
**C1QC_HUMAN**	0,494626	1,188945	Control	Complement C1q subcomponent subunit C OS=Homo sapiens GN=C1QC PE=1 SV=3
**ENPP2_HUMAN**	0,503444	2,148557	Control	Ectonucleotide pyrophosphatase/phosphodiesterase family member 2 OS=Homo sapiens GN=ENPP2 PE=1 SV=3
**FHR1_HUMAN**	0,511048	1,112857	Control	Complement factor H-related protein 1 OS=Homo sapiens GN=CFHR1 PE=1 SV=2
**C4BPB_HUMAN**	0,518113	1,069712	Sepsis	C4b-binding protein beta chain OS=Homo sapiens GN=C4BPB PE=1 SV=1
**THAP4_HUMAN**	0,527682	1,283514	Sepsis	THAP domain-containing protein 4 OS=Homo sapiens GN=THAP4 PE=1 SV=2
**LYSC_HUMAN**	0,531896	2,324872	Sepsis	Lysozyme C OS=Homo sapiens GN=LYZ PE=1 SV=1
**TSP1_HUMAN**	0,539938	1,094635	Control	Thrombospondin-1 OS=Homo sapiens GN=THBS1 PE=1 SV=2
**C163A_HUMAN**	0,540824	1,308847	Sepsis	Scavenger receptor cysteine-rich type 1 protein M130 OS=Homo sapiens GN=CD163 PE=1 SV=2
**LYVE1_HUMAN**	0,558105	1,209595	Sepsis	Lymphatic vessel endothelial hyaluronic acid receptor 1 OS=Homo sapiens GN=LYVE1 PE=1 SV=2
**APOA_HUMAN**	0,560954	2,112056	Sepsis	Apolipoprotein(a) OS=Homo sapiens GN=LPA PE=1 SV=1
**NCHL1_HUMAN**	0,56204	1,160829	Control	Neural cell adhesion molecule L1-like protein OS=Homo sapiens GN=CHL1 PE=1 SV=4
**TENX_HUMAN**	0,565089	2,372156	Sepsis	Tenascin-X OS=Homo sapiens GN=TNXB PE=1 SV=4
**PLTP_HUMAN**	0,58202	1,384861	Sepsis	Phospholipid transfer protein OS=Homo sapiens GN=PLTP PE=1 SV=1
**FA9_HUMAN**	0,58833	1,023277	Sepsis	Coagulation factor IX OS=Homo sapiens GN=F9 PE=1 SV=2
**GELS_HUMAN**	0,606981	1,063752	Control	Gelsolin OS=Homo sapiens GN=GSN PE=1 SV=1
**MBL2_HUMAN**	0,626464	1,114724	Sepsis	Mannose-binding protein C OS=Homo sapiens GN=MBL2 PE=1 SV=2

**PROF1_HUMAN**	0,630268	1,16822	Sepsis	Profilin-1 OS=Homo sapiens GN=PFN1 PE=1 SV=2
**CXCL7_HUMAN**	0,651884	1,042647	Control	Platelet basic protein OS=Homo sapiens GN=PPBP PE=1 SV=3
**GNAL_HUMAN**	0,653712	53,91584	Sepsis	Guanine nucleotide-binding protein G(olf) subunit alpha OS=Homo sapiens GN=GNAL PE=1 SV=1
**FA5_HUMAN**	0,677277	1,183501	Control	Coagulation factor V OS=Homo sapiens GN=F5 PE=1 SV=4
**FETUA_HUMAN**	0,677517	1,100223	Control	Alpha-2-HS-glycoprotein OS=Homo sapiens GN=AHSG PE=1 SV=1
**VTDB_HUMAN**	0,683455	1,056735	Control	Vitamin D-binding protein OS=Homo sapiens GN=GC PE=1 SV=1
**KV301_HUMAN**	0,687513	1,131574	Control	Ig kappa chain V-III region B6 OS=Homo sapiens PE=1 SV=1
**SPN90_HUMAN**	0,69167	1,110617	Control	NCK-interacting protein with SH3 domain OS=Homo sapiens GN=NCKIPSD PE=1 SV=1
**CD44_HUMAN**	0,698862	1,185821	Sepsis	CD44 antigen OS=Homo sapiens GN=CD44 PE=1 SV=3
**PROP_HUMAN**	0,699822	1,456781	Sepsis	Properdin OS=Homo sapiens GN=CFP PE=1 SV=2
**CHLE_HUMAN**	0,720798	1,092708	Sepsis	Cholinesterase OS=Homo sapiens GN=BCHE PE=1 SV=1
**CO5_HUMAN**	0,726164	1,001785	Control	Complement C5 OS=Homo sapiens GN=C5 PE=1 SV=4
**MMP9_HUMAN**	0,734534	1,934791	Sepsis	Matrix metalloproteinase-9 OS=Homo sapiens GN=MMP9 PE=1 SV=3
**AMPN_HUMAN**	0,744489	1,295543	Sepsis	Aminopeptidase N OS=Homo sapiens GN=ANPEP PE=1 SV=4
**PF4V_HUMAN**	0,74592	1,474981	Sepsis	Platelet factor 4 variant OS=Homo sapiens GN=PF4V1 PE=1 SV=1
**IC1_HUMAN**	0,748187	1,085942	Sepsis	Plasma protease C1 inhibitor OS=Homo sapiens GN=SERPING1 PE=1 SV=2
**FCG3B_HUMAN**	0,758483	4,847661	Sepsis	Low affinity immunoglobulin gamma Fc region receptor III-B OS=Homo sapiens GN=FCGR3B PE=1 SV=2
**APOA2_HUMAN**	0,768877	1,011682	Control	Apolipoprotein A-II OS=Homo sapiens GN=APOA2 PE=1 SV=1
**ADCY9_HUMAN**	0,791803	1,001436	Sepsis	Adenylate cyclase type 9 OS=Homo sapiens GN=ADCY9 PE=1 SV=4
**CCNT2_HUMAN**	0,802259	1,019297	Control	Cyclin-T2 OS=Homo sapiens GN=CCNT2 PE=1 SV=2
**LUM_HUMAN**	0,818024	1,013299	Control	Lumican OS=Homo sapiens GN=LUM PE=1 SV=2
**MMP2_HUMAN**	0,840501	1,187508	Sepsis	72 kDa type IV collagenase OS=Homo sapiens GN=MMP2 PE=1 SV=2
**C1RL_HUMAN**	0,853469	1,021289	Control	Complement C1r subcomponent-like protein OS=Homo sapiens GN=C1RL PE=1 SV=2
**FBLN3_HUMAN**	0,853607	1,072444	Sepsis	EGF-containing fibulin-like extracellular matrix protein 1 OS=Homo sapiens GN=EFEMP1 PE=1 SV=2
**CO2_HUMAN**	0,870777	1,082154	Sepsis	Complement C2 OS=Homo sapiens GN=C2 PE=1 SV=2
**ZA2G_HUMAN**	0,889758	1,017049	Sepsis	Zinc-alpha-2-glycoprotein OS=Homo sapiens GN=AZGP1 PE=1 SV=2
**SAMP_HUMAN**	0,907149	1,072198	Control	Serum amyloid P-component OS=Homo sapiens GN=APCS PE=1 SV=2
**PROZ_HUMAN**	0,960429	1,923432	Control	Vitamin K-dependent protein Z OS=Homo sapiens GN=PROZ PE=1 SV=2
**ATRN_HUMAN**	0,970548	1,028294	Sepsis	Attractin OS=Homo sapiens GN=ATRN PE=1 SV=2
**ZSC29_HUMAN**	0,970879	1,32036	Sepsis	Zinc finger and SCAN domain-containing protein 29 OS=Homo sapiens GN=ZSCAN29 PE=1 SV=2
**LDHB_HUMAN**	0,988792	1,184816	Control	L-lactate dehydrogenase B chain OS=Homo sapiens GN=LDHB PE=1 SV=2
**MED23_HUMAN**			---	Mediator of RNA polymerase II transcription subunit 23 OS=Homo sapiens GN=MED23 PE=1 SV=2

Table S2. Proteins identified with at least two peptides and an ANOVA p-value ≤ 0.05 and a ratio>2 in either direction.

Accession	Anova (p)	Max fold change	Highest mean condition	Description
LBP_HUMAN	2,60E-09	29,31263	Sepsis	Lipopolysaccharide-binding protein OS=Homo sapiens GN=LBP PE=1 SV=3
A2GL_HUMAN	3,20E-08	4,426432	Sepsis	Leucine-rich alpha-2-glycoprotein OS=Homo sapiens GN=LRG1 PE=1 SV=2
CRP_HUMAN	8,14E-08	30,41798	Sepsis	C-reactive protein OS=Homo sapiens GN=CRP PE=1 SV=1
SAA2_HUMAN	7,47E-07	84,27766	Sepsis	Serum amyloid A-2 protein OS=Homo sapiens GN=SAA2 PE=1 SV=1
IPSP_HUMAN	1,21E-06	5,010882	Control	Plasma serine protease inhibitor OS=Homo sapiens GN=SERPINA5 PE=1 SV=3
FINC_HUMAN	9,13E-06	8,758802	Control	Fibronectin OS=Homo sapiens GN=FN1 PE=1 SV=4
HBB_HUMAN	1,11E-05	25,27837	Sepsis	Hemoglobin subunit beta OS=Homo sapiens GN=HBB PE=1 SV=2
AACT_HUMAN	1,88E-05	5,283651	Sepsis	Alpha-1-antichymotrypsin OS=Homo sapiens GN=SERPINA3 PE=1 SV=2
SAA1_HUMAN	2,97E-05	76,09709	Sepsis	Serum amyloid A-1 protein OS=Homo sapiens GN=SAA1 PE=1 SV=1
HBA_HUMAN	3,78E-05	16,39969	Sepsis	Hemoglobin subunit alpha OS=Homo sapiens GN=HBA1 PE=1 SV=2
CATA_HUMAN	5,92E-05	7,286451	Sepsis	Catalase OS=Homo sapiens GN=CAT PE=1 SV=3
HABP2_HUMAN	0,0001	2,179075	Control	Hyaluronan-binding protein 2 OS=Homo sapiens GN=HABP2 PE=1 SV=1
TTHY_HUMAN	0,0002	2,365905	Control	Transthyretin OS=Homo sapiens GN=TTR PE=1 SV=1
CAH1_HUMAN	0,0002	8,950114	Sepsis	Carbonic anhydrase 1 OS=Homo sapiens GN=CA1 PE=1 SV=2
CFAB_HUMAN	0,0003	2,048895	Sepsis	Complement factor B OS=Homo sapiens GN=CFB PE=1 SV=2
LYAM1_HUMAN	0,0004	2,675523	Sepsis	L-selectin OS=Homo sapiens GN=SELL PE=1 SV=2
NGAL_HUMAN	0,0005	7,169257	Sepsis	Neutrophil gelatinase-associated lipocalin OS=Homo sapiens GN=LCN2 PE=1 SV=2
B2MG_HUMAN	0,0007	3,486171	Sepsis	Beta-2-microglobulin OS=Homo sapiens GN=B2M PE=1 SV=1
FA12_HUMAN	0,0008	3,237085	Control	Coagulation factor XII OS=Homo sapiens GN=F12 PE=1 SV=3
CD14_HUMAN	0,0008	2,784851	Sepsis	Monocyte differentiation antigen CD14 OS=Homo sapiens GN=CD14 PE=1 SV=2
FIBG_HUMAN	0,0009	5,317049	Sepsis	Fibrinogen gamma chain OS=Homo sapiens GN=FGG PE=1 SV=3
TRFL_HUMAN	0,0011	9,346009	Sepsis	Lactotransferrin OS=Homo sapiens GN=LTF PE=1 SV=6
ITIH3_HUMAN	0,0012	2,192084	Sepsis	Inter-alpha-trypsin inhibitor heavy chain H3 OS=Homo sapiens GN=ITIH3 PE=1 SV=2
PRDX2_HUMAN	0,0018	7,267809	Sepsis	Peroxiredoxin-2 OS=Homo sapiens GN=PRDX2 PE=1 SV=5
A1AG1_HUMAN	0,0020	2,94118	Sepsis	Alpha-1-acid glycoprotein 1 OS=Homo sapiens GN=ORM1 PE=1 SV=1
FIBB_HUMAN	0,0021	7,926361	Sepsis	Fibrinogen beta chain OS=Homo sapiens GN=FGB PE=1 SV=2
APOA4_HUMAN	0,0022	3,062538	Control	Apolipoprotein A-IV OS=Homo sapiens GN=APOA4 PE=1 SV=3
RET4_HUMAN	0,0023	2,804231	Control	Retinol-binding protein 4 OS=Homo sapiens GN=RBP4 PE=1 SV=3
PLMN_HUMAN	0,0024	2,124858	Control	Plasminogen OS=Homo sapiens GN=PLG PE=1 SV=2
SHBG_HUMAN	0,0048	2,27438	Sepsis	Sex hormone-binding globulin OS=Homo sapiens GN=SHBG PE=1 SV=2
SPRC_HUMAN	0,0057	2,146585	Control	SPARC OS=Homo sapiens GN=SPARC PE=1 SV=1
FETUB_HUMAN	0,0078	2,061183	Control	Fetuin-B OS=Homo sapiens GN=FETUB PE=1 SV=2

HPT_HUMAN	0,0080	9,390239	Sepsis	Haptoglobin OS=Homo sapiens GN=HP PE=1 SV=1
PTX3_HUMAN	0,0084	8,831504	Sepsis	Pentraxin-related protein PTX3 OS=Homo sapiens GN=PTX3 PE=1 SV=3
6PGD_HUMAN	0,0085	3,129853	Sepsis	6-phosphogluconate dehydrogenase, decarboxylating OS=Homo sapiens GN=PGD PE=1 SV=3
ZPI_HUMAN	0,0112	2,79504	Sepsis	Protein Z-dependent protease inhibitor OS=Homo sapiens GN=SERPINA10 PE=1 SV=1
PLSL_HUMAN	0,0168	2,812893	Sepsis	Plastin-2 OS=Homo sapiens GN=LCP1 PE=1 SV=6
ACTB_HUMAN	0,0199	3,024146	Sepsis	Actin, cytoplasmic 1 OS=Homo sapiens GN=ACTB PE=1 SV=1
POSTN_HUMAN	0,0236	2,072017	Sepsis	Periostin OS=Homo sapiens GN=POSTN PE=1 SV=2
CBPN_HUMAN	0,0262	2,402578	Sepsis	Carboxypeptidase N catalytic chain OS=Homo sapiens GN=CPN1 PE=1 SV=1
APOC1_HUMAN	0,0268	2,093199	Control	Apolipoprotein C-I OS=Homo sapiens GN=APOC1 PE=1 SV=1
LDHA_HUMAN	0,0291	2,579157	Sepsis	L-lactate dehydrogenase A chain OS=Homo sapiens GN=LDHA PE=1 SV=2
CD5L_HUMAN	0,0332	6,760591	Control	CD5 antigen-like OS=Homo sapiens GN=CD5L PE=1 SV=1
ALDOB_HUMAN	0,0372	5,740726	Sepsis	Fructose-bisphosphate aldolase B OS=Homo sapiens GN=ALDOB PE=1 SV=2
